# Extended lymphadenectomy for locally advanced and recurrent rectal cancer

**DOI:** 10.1007/s00384-016-2711-6

**Published:** 2017-01-27

**Authors:** Panagiotis A. Georgiou, S. Mohammed Ali, Gina Brown, Shahnawaz Rasheed, Paris P. Tekkis

**Affiliations:** 1 Department of Colorectal Surgery, The Royal Marsden Hospital NHS Foundation Trust, Fulham Road, SW3 6JJ London, UK; 2 Department of Surgery and Cancer, Academic Surgery, Imperial College, 3rd Floor, Chelsea and Westminster Hospital Campus, Fulham Road, SW10 9NH London, UK; 3 Department of Radiology, The Royal Marsden NHS Foundation Trust, London, UK

**Keywords:** Lateral, Pelvic sidewall, Extended, Lymphadenectomy, Recurrent, Advanced, Locally, Rectal cancer, Exenterative surgery

## Abstract

**Purpose:**

The purpose of this study is to assess the value of extended (lateral) lymphadenectomy (EL) in the operative management of locally advanced and recurrent rectal cancer.

**Methods:**

Patients that underwent exenterative surgery for locally advanced or recurrent rectal cancer between 2006 and 2009 were included in the study. A decision for EL was taken at the local multidisciplinary meeting based on the radiological findings. Perioperative and oncological outcomes were assessed and compared between the EL and non-EL group prospectively.

**Results:**

Forty-one consecutive patients were included in the study (EL = 17). The median age was 57 (40–71) for EL and 66 (39–81) years for non-EL. Of patients, 27 (EL = 13) and 14 (EL = 4) underwent pelvic exenteration and abdominosacral resection, respectively. Twelve (EL = 7) patients were diagnosed with locally advanced primary rectal cancer. Thirty-one (EL = 12) patients received neoadjuvant radiotherapy. The median intraoperative time, blood loss and hospital stay were 9 h (3–13), 1.5 l (0.3–7) and 14 days (12–72), respectively, for the EL group, and 8 h (4–15), 1.6 l (0.25–17) and 14 days (10–86), respectively, for the non-EL (*p* ≥ 0.394). Morbidity was similar between the two groups (EL = 4, non-EL = 9; *p* = 0.344). Complete tumour resection (R0) was achieved in 30 (73.17%) patients, 12 (70.58%) in the EL group and 18 (75%) in the non-EL group (*p* = 0.649). There was no significant difference in 5-year survival (EL = 60.7%, non-EL = 75.2%; *p* = 0.447), local recurrence (EL = 53.6%, non-EL = 65.4%; *p* = 0.489) and disease-free survival (EL = 53.6%, non-EL = 51.4%; *p* = 0.814).

**Conclusions:**

The present study demonstrated that EL does not provide a statistically significant advantage in survival or recurrence rates, for patients with locally advanced primary or recurrent rectal cancer.

## Introduction

The presence of lateral pelvic lymph node metastases in rectal cancer was first reported in the 1950s [1]. Nodal involvement has subsequently been demonstrated to adversely affect the prognosis [2–7], resulting in higher incidence of local recurrence and reduced survival [2, 3, 5, 6]. Lateral pelvic lymph node metastases (along the obturator, internal iliac and medial aspect of the external iliac artery) have been reported to be involved in 10–25% of patients operated for rectal cancer [1–3]. Based on these findings, surgeons in Japan have adopted the technique of lateral pelvic sidewall (extended) lymphadenectomy (EL) to supplement conventional rectal surgery, aiming to reduce local recurrence rates and improve the cancer-specific survival [8–10].

There has been an on-going debate about the value of lateral pelvic sidewall lymphadenectomy in the management of primary rectal cancer. A recent meta-analysis [11] demonstrated a higher likelihood of poor quality of life in terms of sexual and urinary dysfunction in patients undergoing extended lymphadenectomy, without any significant oncological benefit except in stage III low rectal cancers. The authors concluded that the value of lateral pelvic sidewall should be further investigated in the setting of a randomised controlled study. It was felt that extended lymphadenectomy should be compared to neoadjuvant chemoradiotherapy, which has been shown to decrease the risk of local recurrence by up to 61% [12] although without any significant improvement in the overall survival.

In a series of 93 patients who underwent a pelvic exenteration for locally advanced rectal cancer [13], it was demonstrated that lateral pelvic sidewall lymph node involvement was significantly associated with reduced survival (*p* = 0.01) compared to patients without any lymph node metastases.

Lateral local recurrence (including lateral pelvic sidewall lymph node involvement) following surgery for rectal cancer has been reported to be about 27% of all patients who present with local recurrence [14, 15]. It is, therefore, a common form of local failure that requires aggressive management either in the form of chemoradiotherapy or excision of the lymph nodes. Failure to address this may result in disease progression within the adjacent iliac vessels or the sciatic nerve increasing the likelihood of the disease becoming irresectable [16].

In the group of patients with locally advanced primary and recurrent rectal cancers that require exenterative pelvic surgery with curative intent, the value of extended lymphadenectomy has not been investigated. To the authors’ knowledge, this is the first study that aims to assess the oncological benefit of performing lateral sidewall pelvic lymphadenectomy in patients who undergo surgery beyond the boundaries of a standard total mesorectal excision (TME).

## Methods

### Study design and subjects

Data were extracted from a prospective database of consecutive patients who underwent exenterative pelvic surgery for locally advanced primary and recurrent rectal cancer between March 2006 and October 2009. Routine data recorded in the database included (1) patient demographics; (2) operative details; (3) neoadjuvant treatment and staging; (4) tumour histopathology and (5) follow-up data, including the cause of death when applicable and local recurrence data. Data were collected in a modified Microsoft® Excel/SPSS spreadsheet. The decision for lateral pelvic sidewall lymphadenectomy was taken either during the local multidisciplinary meetings based on radiological imaging evidence of sidewall nodes involved by tumour or intraoperative evidence of lateral pelvic sidewall lymph node metastasis. This was an observational study without any influence on the management of patients and it was approved by the regional cancer network.

### Inclusion and exclusion criteria

All adult patients (age >18 years) undergoing pelvic exenterative surgery for locally advanced primary or recurrent rectal cancer were included. Patients with metastatic cancer (TNM Stage IV) or those undergoing palliative procedures were excluded.

### End points and outcomes

The principle end points were local recurrence of rectal cancer, diagnosed by radiological or histopathological examination and cancer-specific survival (CSS). Perioperative outcomes such as intraoperative time, estimated blood loss, length of stay and morbidity were assessed between the EL and non-EL groups. The intraoperative time was defined as the length of time for surgery and did not include anaesthetic time. Similarly, estimated blood loss was recorded in litres lost during surgery. Length of stay was defined as the time taken to discharge from the first postoperative day. Morbidity was defined as any unexpected event that deviated from the ideal postoperative recovery that either delayed discharge, required treatment with antibiotics, additional invasive procedures (e.g. percutaneous drainage) or further surgery.

### Extended lymphadenectomy technique

Following dissection and mobilisation of the sigmoid and descending colon, the inferior mesenteric artery and vein were ligated and divided high (in primary resections and recurrent operations if not done previously). The small bowel was reflected and protected to allow exposure of the pelvis to enable the initiation of the lateral dissection. The retroperitoneum was dissected at the level of the bifurcation of the aorta exposing the origin of the common iliac arteries. The lateral lymph node dissection was performed the same way as it was previously described in 1989 by Moriya [10]. The bifurcation of the common iliac artery was identified and the internal iliac artery was traced down towards the lower pelvis. The lateral pelvic lymphadenectomy was performed either en bloc with the internal iliac vessels or by preserving them, depending on the proximity of tumour to the internal iliac artery. This decision was often guided by preoperative assessment at the local colorectal multidisciplinary team meeting.

The dissection of the lateral pelvic sidewall lymph nodes commenced with the dissection of the internal iliac vessels from the medial structures. The branches of the internal iliac vessels were identified, ligated and divided as close to their origin as possible. For the cases that the tumour was close to the iliac artery, the lateral pelvic sidewall lymphadenectomy was performed en bloc with the resected specimen including the unilateral internal iliac artery and preservation of the superior vesical artery and the obturator nerve, if possible.

When the lateral pelvic sidewall lymphadenectomy was performed to remove lesions suspicious for malignancy or isolated malignant lateral lymph nodes, the nerve preserving approach was utilised (conventional lateral dissection) [10]. The specimen was resected en bloc with the tumour while the lateral pelvic sidewall spaces were opened between the lateral aspect of the internal iliac vessels and the pelvic sidewall, exposing the lateral lymphatic tissue and enabling the harvesting of lymph nodes. Using the nerve preserving technique, the obturator nerve and internal iliac vessels with their branches were preserved without disturbing the sacral nerve plexuses.

With the completion of the lateral pelvic sidewall dissection and the abdominal mobilisation of the specimen, depending on the extent of the tumour, a pelvic exenterative procedure was performed with or without the removal of the sacrum (abdominosacral resection).

### Statistics

Categorical demographic characteristics were compared using chi-squared test; age and continuous variables were compared using ANOVA. Survival curves were obtained using the Kaplan-Meier method, which allows for estimation of outcomes in datasets in which censored data are present. The log-rank test was used to compare the two groups. Censored patients were considered those who had incomplete follow-up or those who died during follow-up before reaching 5 years without experiencing the outcomes of interest. Local recurrence and disease-free survival curves were estimated in relation to both EL and non-EL groups. Comparisons were considered statistically significant at a probability value of *p* < 0.05.

Data were entered in the Statistical Package for Social Sciences (SPSS Inc., Chicago, IL), which was also used for the statistical analysis.

## Results

### Demographic data (Table 1)

A total of 41 (28 males) patients with a median age of 64 (39–81) were included in the present study. Twelve (29.27%) patients with locally advanced primary and 29 (70.73%) patients with recurrent rectal cancer underwent exenterative pelvic surgery. Lateral pelvic sidewall lymphadenectomy was performed in 17 (41.4%) patients. The patients in the EL group were younger (median age 57 (40–71)) compared to the non-EL group (median 66 (39–81); *p* = 0.042). There was no statistically significant difference in the gender distribution between the two groups (*p* = 0.467). Thirty-one (75.6%) patients were treated with neoadjuvant radiotherapy; 12 and 19 were in the EL and non-EL groups, respectively. This difference did not reach statistical significance (*p* = 0.529). All patients had open surgery with 14 (34.15%) of them undergoing an abdominosacral resection with the remainder having other pelvic exenterative procedures. There was no statistically significant difference in the type of surgery that was performed in the two groups (*p* = 0.192). Four plastic reconstructions with flaps were performed in EL group and 13 in the non-EL group. This difference approached statistical significance (*p* = 0.051).

**Table 1:** Demographic data

	Total (*n* = 41)	EL (*n* = 17)	Non-EL (*n* = 24)	*p*
Age (mean, SD)	64 (39–81)	57 (40–71)	66 (39–81)	0.042
Sex (M/F)	28/13	11/6	17/7	0.678
Recurrent cancer	29	10	19	0.158
Primary cancer	12	7	5	
Radiotherapy	31	12	19	0.529
Surgery				0.228
ASR	14	4	10	
PE	27	13	14	
Flaps	17	4	13	0.051
Histopathology type				0.791
Adenocarcinoma	37	15	22	
Signet cell	2	1	1	
Differentiation				0.636
Well/moderate	20	7	13	
Poor/mucinous	19	9	10	
PNI	19	6	13	0.242
Vascular invasion	20	7	13	0.433

*n* number, *EL* extended lymphadenectomy, *non-EL* non-extended lymphadenectomy, *p* probability of obtaining a test statistic at least as extreme as the one that was actually observed, *M* male, *RT* radiotherapy, *F* female, *PE* pelvic exenteration, *APER* abdominoperineal excision of the rectum, *SCC* squamous cell carcinoma, *PNI* perineural invasion

### Perioperative outcomes (Table 2)

The median intraoperative time (IOT) was 9 (3–13) and 8 h (4–15) for the EL and non-EL groups, respectively. The median intraoperative blood loss was 1.5 (0.3–7) and 1.6 l (0.25–17) for the EL and non-EL groups. The median length of stay was 15 days for both groups. Neither of the above perioperative outcomes reached statistical significance (*p* ≥ 0.344). A total of 4/17 (23.52) complications were observed in the EL group and 9/24 (37.5%) in the non-EL group. This difference was not statistically significant between the two groups either (*p* = 0.344). For the EL group, there were two patients with Clavien-Dindo [17] grade 4 complications and two with grade 3. For Non-EL group, there were one grade 4, five grade 3, two grade 2 and one grade 1. There was no significance between the two groups (*p* = 0.367). There was no perioperative mortality.

**Table 2:** Perioperative outcomes

	Total (*n* = 41)	EL (*n* = 17)	Non-EL (*n* = 24)	*p*
IOT (median, range)	8 (3–15)	9 (3–13)	8 (4–15)	0.903
Blood loss (median, range)	1.6 (0.25–17)	1.5 (0.3–7)	1.6 (0.25–17)	0.394
LOS (median, range)	15 (10–86)	15 (12–72)	14 (10–86)	0.887
Morbidity	13	4	9	0.344

*n* number, *EL* extended lymphadenectomy, *non-EL* non-extended lymphadenectomy, *IOT* intraoperative time, *LOS* length of stay, *EL* extended lymphadenectomy, *ASR* abdominosacral resection

### Oncological outcomes (Table 3)

Curative resections were achieved in 30 (73.17%) patients: 12 (70.58%) in the EL group and 18 (75%) in the non-EL group. There were 8 (19.51%) patients with microscopic residual disease (R1) at the resection margins, 3 (17.64%) in the EL group and 5 (20.83%) in the non-EL group. In 3 (7.32%) patients, there was macroscopic residual disease at the resection margins (R2), 2 (11.76%) in the EL and 1 (4.17%) in the non-EL group. There was no statistically significant difference between the two groups in terms of tumour clearance (*p* = 0.649). There were 6 (35.29%) patients with malignant lateral pelvic sidewall lymph nodes. Other histopathological data are shown in Table 1. There was no significant difference between the two groups in terms of the histopathology either (*p* ≥ 0.292). Two patients responded fully to neoadjuvant radiotherapy, one in each group.

**Table 3:** Oncological outcomes

	Total (*n* = 41)	EL (*n* = 17)	Non-EL (*n* = 24)	*p*
Clearance				0.649
R0	30 (73.17%)	12 (70.58%)	18 (75%)	
R1	8 (19.51%)	3 (17.64%)	5 (20.83%)	
R2	3 (7.32%)	2 (11.76%)	1 (4.17%)	
Local recurrence	12/38 (31.58%)	6/15 (40%)	6/23 (26.08%)	0.367
Distant recurrence	8 (19.51%)	2 (11.76%)	6 (25%)	0.292

*n* number, *EL* extended lymphadenectomy, *non-EL* non-extended lymphadenectomy, *R0* complete tumour resection, *R1* microscopic residual tumour at the margins, *R2* macroscopic residual tumour at the margins or irresectable tumour, *LR* local recurrence, *DR* distant recurrence

Since three patients had R2 resections, 38 patients were included for recurrence and disease-free survival. There were 6 patients in each group that were diagnosed with local recurrence following potentially curative surgery (R0 and R1). There was no statistically significant difference between the two groups (*p* = 0.367). All local recurrences were diagnosed within 3 years from surgery. There were two local recurrences per year in the EL group (Table 4). There were four local recurrences within the first year of surgery in the non-EL group, one in the second and one in the third. Distant metastases were diagnosed in two (13.33%) and six (26.08%) patients in the EL and non-EL groups, respectively (all patients). This difference did not reach statistical significance (*p* = 0.292).

**Table 4:** Recurrences diagnosed per year

	1 year	2 years	3 years	5 years
Local recurrence				
EL	2	2	2	0
Non-EL	4	1	1	0
Distant recurrence				
EL	1	1	0	0
Non-EL	4	1	1	0

*EL* extended lymphadenectomy, *non-EL* non-extended lymphadenectomy

**Fig. 1:**
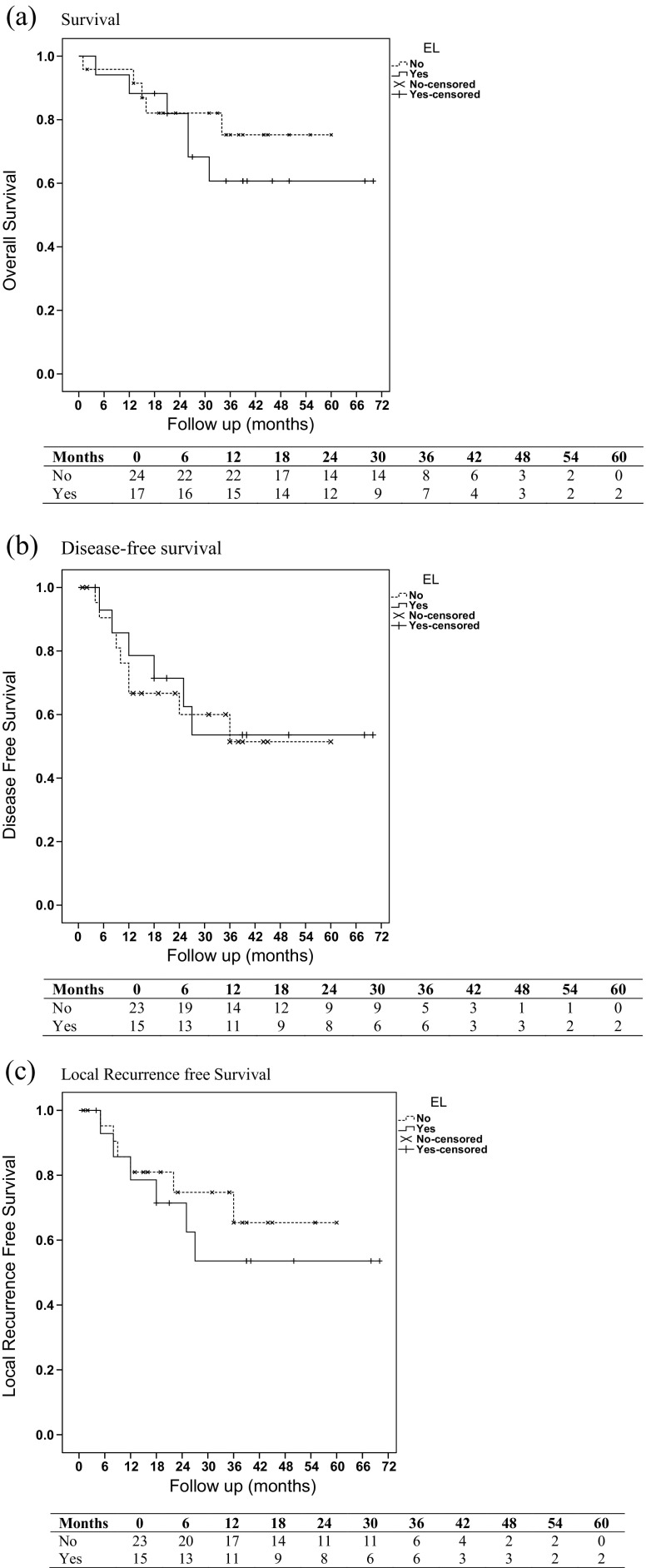
Survival curves. **a** Kaplan-Meier Survival curve—overall survival. **b** Kaplan-Meier Survival curve—disease-free survival. **c** Kaplan-Meier Survival curve—local recurrence-free survival. *EL* extended lymphadenectomy

Overall survival, disease-free survival and local recurrence-free survival curves were generated using the Kaplan-Meier method (Fig. 1). The two groups were compared using the log-rank test. One (1/41; 2.44%) patient in the non-EL group was lost to follow-up 2 months after curative surgery (R0). The longest follow-up was 70 months with median follow-up of 33 months. For overall survival, (Fig. 1a) 88.2% (SE = 7.8%), 81.9% (SE = 9.5%) and 60.7% (SE = 12.7%) patients were alive at 12, 24 and 36 months, respectively, following surgery in the EL group; 95.8% (SE = 4.1%), 82.1% (SE = 8.2%) and 75.2% (SE = 9.9%) patients were alive at 12, 24 and 36 months, respectively, in the non-EL group. There was no statistically significant difference between the two groups (*p* = 0.447).

For the disease- and local recurrence-free survival curves, patients with either complete (R0) or residual microscopic disease at the margins (R1) were included. A total of 78.6% (SE = 11%), 71.4%(SE = 12.1%) and 53.6% (SE = 14.2%) were disease free at 12, 24 and 36 months from surgery in the EL group, respectively; 66.7% (SE = 10.3%), 60% (SE = 11.2%) and 51.4% (SE = 12.5%) were disease free at 12, 24 and 36 months following surgery in the non-EL group, respectively (Fig. 1b). This difference did not achieve statistical significance (*p* = 0.814).

A total of 78.6% (SE = 11%), 71.4% (SE = 12.1%) and 53.6% (SE = 14.2%) were local recurrence-free at 12, 24 and 36 months from surgery, respectively, in the EL group; 81% (SE = 8.6%), 74.7% (SE = 9.9%) and 65.4% (SE = 12.3%) were local recurrence-free after 12, 24 and 36 months, respectively, following surgery in the non-EL group (Fig. 1c). This difference did not reach statistical significance (*p* = 0.489).

## Discussion

This was an observational study that investigated the value of lateral pelvic sidewall lymphadenectomy in a group of consecutive patients who underwent exenterative surgery for locally advanced primary and recurrent rectal cancer. The present study demonstrated no statistically significant differences between EL and non-EL regarding perioperative (intraoperative time and blood loss, morbidity and length of hospital stay) and oncological (clearance rates, recurrences, overall and disease-free survival) outcomes. However, the patients in the EL group were younger than the patients in the non-EL group.

Intraoperative time and blood loss always have concerned surgeons when planning extended lymphadenectomy in primary rectal cancer patients. Intraoperative time and blood loss have been previously shown to increase by 76 min and 500 ml, respectively, when lateral pelvic sidewall lymphadenectomy was performed for primary rectal cancer [11]. The present study investigated the value of EL in patients that underwent exenterative pelvic surgery for locally advanced primary and recurrent rectal cancer and demonstrated no substantial difference between the two groups. Exenterative pelvic surgery alone is known to last longer and to result in significant intraoperative blood loss [16, 13]. This is reflected by the results of this study and may explain the absence of significant difference between the two groups.

Overall survival, local recurrence-free and disease-free survival rates were demonstrated to have no significant difference between the EL and non-EL group. However, the difference in the overall and local recurrence-free survival was large and cannot be overlooked on the basis of statistical significance, with the non-EL group having a more favourable outcome. This may be a reflection of a more advanced and challenging to treat disease, for the patients with tumour invading the lateral pelvic sidewall. These findings need to be interpreted with caution though due to the small size of this study. A prospective randomised study with a larger number of patients may provide the power to answer this question. All recurrences occurred within 3 years following surgery which is consistent with the results published by other studies that report the majority of the recurrences were diagnosed within 3 years from surgery [18, 19] although tumours may recur up to 10 years following surgery [19].

Neoadjuvant (chemo)radiotherapy has been shown to reduce local failure significantly following surgery for recurrent rectal cancer (*p* = 0.036) but has not been demonstrated to influence overall survival and metastasis-free survival [20]. For primary rectal cancer, published studies suggested that radiotherapy might have a similar result to EL in regards to overall and disease-free survival [21, 22]. For recurrent and locally advanced rectal cancers, the evidence is not clear with regards to the value of radiotherapy. At the authors’ Institute, neoadjuvant chemoradiotherapy is always used for patients that undergo exenterative surgery for primary and recurrent rectal cancer when it is not contraindicated. It is also of high importance to emphasise that further research investigating the role of chemoradiotherapy is essential in this group of patients.

Lateral pelvic sidewall lymphadenectomy has an adverse impact on the quality of life of patients, in terms of urinary [23–25] and sexual [21, 26, 27] function as previously demonstrated for primary rectal cancer, as the autonomic nerves in the region are at high risk or are sacrificed to achieve the optimum oncological outcome. Therefore, a significant amount of time is required preoperatively to discuss the benefits and risks of extended lymphadenectomy with patients due to undergoing surgery for primary cancer surgery. The quality of life of patients undergoing exenterative surgery has also been demonstrated to be adversely affected [28]. This is due to the sacrifice of the nerves along with other intrapelvic organs due to their close relationship or proximity to the tumour. Therefore, en bloc dissection of the lateral pelvic lymph nodes may be preferred to nerve preservation lymphadenectomy as it very rarely influences the postoperative quality of life in this group of patients. A recent study [29] that aimed to measure the quality of life for longer-term disease-free survivors after pelvic exenteration demonstrated comparable results to low anterior resection or abdominoperineal excision of the rectum for primary rectal cancer. They showed a low score for the physical component but similar scores in the mental component of the scoring form.

In the present study, the patients that underwent lateral pelvic sidewall lymphadenectomy were younger than the patients who did not. However, age is not a factor in the decision process to perform this procedure. When a patient is deemed fit for exenterative surgery, it is extremely unlikely that extended lymphadenectomy will be rejected as an option, if required. Exenterative pelvic surgery carries significant risks with the morbidity ranging up to more than 50% of the operated patients [30–32, 18, 16]. Therefore, patients are thoroughly examined and tested to ensure their fitness to endure the operation and minimise morbidity.

A decision for lateral pelvic sidewall lymphadenectomy was taken in this series of patients when there was either radiological or intraoperative evidence of malignant lateral pelvic sidewall lymph nodes, or the tumour invaded the lateral pelvic compartment and had to be removed en bloc with the lymph nodes. Tumours within the lateral pelvic compartment are considered to have poorer prognosis [13, 18, 19]. The fact that younger patients had to undergo lateral pelvic sidewall lymphadenectomy may indicate that their tumour was more aggressive, a factor that may need to be taken into account when considering young patients for exenterative pelvic surgery. However, further studies investigating the tumour histopathology of these patients are essential to conclude this.

Lateral compartment recurrences, including the lateral lymph nodes, can be up to 26.7% of the patterns of local recurrence [33, 14] and demonstrated to be a factor that adversely affects survival following surgery [14, 33]. This can be partially a result of the high risk of incomplete tumour resection when it extends within the lateral pelvic compartment. Moore et al. [18] demonstrated that pelvic sidewall recurrence was a strong determinant of incomplete resection (*p* = 0.004). Similarly, Sagar et al. [19], in a series of 40 patients that underwent abdominosacral resection for recurrent rectal cancer, demonstrated that 15/20 (75%) patients with non-curative resections had lateral compartment recurrence. This led them to consider extending their resection margins in future cases when preoperative MR imaging identifies tumour within the lateral compartment. Performing lateral pelvic sidewall lymphadenectomy might be a method of extending the lateral resection margins and may be considered in patients when aiming curative resection.

One of the strengths of the present study was that the patients were managed and operated by the same multidisciplinary team thus eliminating bias. One of the limitations of this study is the relatively small number of patients that were included requiring a careful interpretation of the results of this study. Exenterative surgery or this group of patients is performed on about 250 patients a year in the UK and several years will be required to gather adequate data to increase the power of the study significantly. A second limitation may be that a single colorectal surgeon, albeit with experience in pelvic cancer surgery, performed these procedures. Thus, the reported outcomes might not be representative since there is likely to be some variance between surgeons.

## Conclusion

The present study demonstrated that lateral pelvic sidewall lymphadenectomy does not offer a statistically significant advantage regarding the oncological and perioperative outcomes in patients that undergo exenterative surgery for locally advanced primary and recurrent rectal cancer, and that it is safe to perform. However, the study size is small, and therefore these results should be interpreted with caution.
